# A data review of airway management in patients with oral cavity or oropharyngeal cancer: a single-institution experience

**DOI:** 10.1186/s12871-019-0770-2

**Published:** 2019-06-01

**Authors:** Gang Zheng, Lei Feng, Carol M. Lewis

**Affiliations:** 1 Department of Anesthesiology and Perioperative Medicine, The University of Texas MD Anderson Cancer Center, 1515 Holcombe Boulevard, Faculty Center – Unit 409, Houston, TX 77030 USA; 2 Department of Biostatistics, The University of Texas MD Anderson Cancer Center, FCT4.5047, T. Boone Pickens Academic Tower, 1400 Pressler St, Houston, TX 77030-4008 USA; 3 Department of Head and Neck Surgery, The University of Texas MD Anderson Cancer Center, Unit 1445, T. Boone Pickens Academic Tower, 1515 Holcombe Blvd, Houston, TX 77030-4009 USA

**Keywords:** Radiotherapy, Cancer, Trismus, Upper aerodigestive track, Airway

## Abstract

**Background:**

Oral cavity and oropharyngeal cancer impose significant threat to airway management. Head and neck radiotherapy (HNRT) may further increase the difficulty of tracheal intubation. We hypothesized that a history of HNRT would be associated with a high rate of difficult tracheal intubation.

**Methods:**

Adult patients with a history of HNRT were identified. Non-HNRT controls were case-matched by age, sex and body mass index. The tracheal intubation status between the two patient groups (treated vs. untreated with HNRT) was compared. The *t* test was used to evaluate differences in continuous variables between the 2 groups. Fisher’s exact test or a chi-square test was used to test for associations between radiation status and patient characteristics that may be associated with difficult tracheal intubation. Odds ratio and its confidence interval were used to assess the effect of radiation status on intubation status.

**Results:**

The final cohort of 472 matched patients in age, sex and body mass index consisted of 236 patients who had HNRT before surgery and 236 who had upfront surgery without HNRT. The percentage of patients who had restricted neck range of motion in the HNRT group was significantly higher than in the control group (22.3% vs. 11.0%; *p* = 0.001). The proportion of patients with trismus (*p* = 0.11) or difficult tracheal intubation (*p* = 0.73) did not differ significantly between the 2 groups. 12.7% patients in the study had difficult tracheal intubation. Patients who had mallampati scores of 3 or 4 had significantly higher rate of difficult tracheal intubation than did patients with mallampati scores of 1 or 2 (17.8% vs. 8.7%; *p* = 0.004). Multivariate logistic regression model showed no difference between HNRT and intubation status after adjusting neck range of motion and mallampati score (OR = 0.91, 95% CI: 0.510 to1.612).

**Conclusions:**

Previous treatment with HNRT was not associated with additional risk of difficult tracheal intubation. Mallampati score may be a sensitive measurement for difficult tracheal intubation in this patient population.

## Background

In the United States, an estimated 41,000 oral cavity and oropharyngeal malignancies are diagnosed each year [1]. The anatomic sites of oral cavity cancer (OCC) cover the lips, anterior two-thirds of tongue, gingiva, retromolar trigone, buccal mucosa, hard palate and the floor of mouth; whereas, the sites of oropharyngeal cancer (OPC) are located in the posterior one-third of the tongue, palatine or lingual tonsils, soft palate, and posterior pharyngeal wall [2].

With the technologic improvement, radiotherapy, depending the subsite of a disease, has become a primary treatment for many head and neck malignancies in order to maximally maintain functionality of the upper aerodigestive track. Approximately 75% of patients with head and neck squamous cell carcinoma can benefit from head and neck radiation therapy (HNRT) [3, 4]. Although previous receipt of HNRT is a recognized risk factor for difficult airway management, the mechanisms of HNRT- related airway pathologic changes and the overall influences of previous HNRT on the outcomes of tracheal intubation remain unclear.

In this retrospective study, we reviewed the performance and outcomes of tracheal intubation in patients who were undergoing surgery to resect primary OCC or OPC of any stage. The primary goal of the study was to determine if previous HNRT adds additional risk to the airway management in the patients with OCC and OPC disregarding the technique applied. In addition, the factors potentially associated with difficult tracheal intubation (DTI) were also analyzed.

## Methods

### Patient selection

The Institutional Review Board (IRB) at The University of Texas MD Anderson Cancer Center approved this study (IRB No. PA12–0699). The informed consent was waived by IRB because this is a retrospective study. A cancer registry including records of 4011 adult (age ≥ 18 y at the time of surgery) patients with primary diagnoses of OCC or OPC who subsequently underwent resection of the primary OCC or OPC tumor at MD Anderson from 2007 to 2012 was used as the primary study database. Patients who underwent tonsillectomy for positive cervical lymphadenopathy with unclear primary disease, whose cases had been used for teaching of flexible endoscopy for tracheal intubation and patients who were < 18 years of age at the time of HNRT were excluded from this study. In addition, because the majority of the patients in the registry received intensity modulated radiation therapy, those who underwent brachytherapy or proton therapy were excluded in order to ensure the uniformity of the sample.

All the patients in the registry who qualified for the study were assigned into 1 of 2 groups according to whether they had received HNRT: the HNRT group, which consisted of patients who received HNRT before tumor resection, and the non-HNRT group, who underwent upfront resection without having received HNRT. Because some of the data for this study were embedded in the notes describing tracheal intubation in patients’ medical records, requiring a manual search, we used a matching strategy to avoid having to hand-search the entire registry. We first identified the HNRT group as described above; then, we used the exact matching method to select matched controls from the non-HNRT group. The control patients were selected according to age, sex, and body mass index (BMI) to match the patients in the HNRT group at a ratio of 1:1. The matching range for age was ±5 y. For BMI, the 2 groups were matched at 6 levels: ≤ 18.5 kg/m^2^, 18.6–25.0 kg/m^2^, 25.1–30.0 kg/m^2^, 30.1–35.0 kg/m^2^, 35.1–40.0 kg/m^2^ and ≥ 40.1 kg/m^2^. Each patient in the HNRT group was successfully matched with a non-HNRT control patient.

### Data collection

The following data were electronically retrieved from the patients’ medical records: age, sex, BMI, American Society of Anesthesiologists physical status score (ASA score), airway assessment (mouth opening, neck range of motion, edentulous and MP scores), cancer diagnosis, type of surgery, and whether the patient had a history of HNRT. Data on radiotherapy and on the method of tracheal intubation were manually retrieved from the records after matching. The missing data in the primary data sets were manually searched for and placed in the corresponding data set. When a patient had multiple surgeries after HNRT, the data for the first surgery with tracheal intubation after HNRT were used. For airway assessment, most anesthesia providers in our practice considered patients to have trismus if their inter-incisional distance was < 2 finger breadths (typically < 3.5 cm). However, no standardized criteria were used to measure neck extension, so the neck range of motion was based on providers’ subjective judgment. In this study, edentulous referred to a patient with complete upper, lower, or whole-mouth removable dentures. We included the grade of laryngeal view during tracheal intubation in the analysis to reflect the intubation effort according to the Cormack-Lehane system [5]. The grade of laryngeal view mainly applied to the patients who had been intubated via either direct laryngoscopy or video laryngoscopy of any type. For flexible endoscopy trachea intubation, grade I was used for data analysis.

### Statistical analysis

Summary statistics, including mean, standard deviation, median, and range, were calculated for continuous variables, such as age, BMI, and the interval between radiotherapy and surgery. Frequencies and percentages were used to summarize data for categorical variables, such as sex, BMI, Mallampati (MP) score, mouth opening, neck range of motion, cancer stage, radiation status, and airway intubation status. The *t* test was used to evaluate differences in continuous variables between the 2 patient groups. Fisher’s exact test or a chi-square test was used to test for associations between radiation status (HNRT or control) and patient characteristics that may be associated with difficulty of tracheal intubation, including sex, BMI, MP score, mouth opening, neck range of motion, cancer stage, intubation difficulty status (difficult or easy) and patient characteristics. Odds ratio and its confidence interval were used to assess the effect of radiation status on intubation difficulty status. A *P* value of less than 0.05 was considered statistically significant. The statistical software SAS 9.3 (SAS, Cary, NC) was used for all the analyses.

## Results

### Patient characteristics

Of the 4011 records of eligible patients included in the cancer registry database, 3999 records were eligible for inclusion in this study; 8 patients whose procedures had been used for teaching airway management and 4 patients who had received proton therapy were excluded. The final study cohort of 472 matched patients consisted of 236 patients who had HNRT before surgery and 236 who had upfront surgery without HNRT. The mean (± standard deviation) age of the included patients was 58.7 (± 9.1) years, and their mean BMI was 25.5 (± 4.5) kg/m^2^. The mean interval between completion of HNRT and surgery in the HNRT group was 330.3 (± 474.5) days. Eight primary cancer locations were found in 447 patients. The characteristics of the study cohort are detailed in Table 1.

**Table 1:** Characteristics of 472 patients with oral cavity or oropharyngeal cancer

Characteristic	No. of patients	%
Head/neck radiation		
No	236	50.0
Yes	236	50.0
Sex		
Female	60	12.71
Male	412	87.29
Body mass index (kg/m^2^)		
≤ 18.5	17	3.6
18.6–25.0	228	48.3
25.1–30.0	157	33.26
30.1–35.0	51	10.81
35.1–40.0	16	3.39
≥ 40.1	3	0.64
Mallampati score		
Data missing ^a^	16	
1	63	13.82
2	205	44.96
3	130	28.51
4	58	12.72
Mouth opening		
Data missing	3	
Full	367	78.25
Limited	102	21.75
Thyromental distance (≈ cm)		
Data missing	391	
< 5	20	24.69
≥ 5	61	75.31
Edentulous Removable denture		
Data missing	1	
Yes	101	21.44
No	370	78.56
Neck movement		
Data missing	3	
Full range of motion	391	83.37
Restricted	78	16.63
Cancer location		
Tongue	218	46.19
Tonsil	98	20.76
Floor of mouth	49	10.38
Mandible	26	5.51
Retromolar region	24	5.08
Buccal mucosa	22	4.66
Maxilla gingiva	5	1.06
Soft palate	5	1.06
Multiple locations	25	5.30
Cancer stage		
Data missing	3	
T1	84	17.91
T2	155	33.05
T3	92	19.62
VT4	110	23.45
TX	24	5.12
Not staged	4	0.85
Intubation status		
Data missing	16	
Difficult	58	12.72
Easy	398	87.28
Primary intubation technique		
Data missing	10	
Direct laryngoscopy	267	57.79
Asleep FOI	137	29.65
Awake FOI	27	5.84
AirTraq video laryngoscopy	15	3.25
C-MAC video laryngoscopy	7	1.52
LMA intubation	3	0.65
Tracheostomy	6	1.30
Secondary intubation technique		
AirTraq video laryngoscopy	4	50
Asleep FOI	4	50

Abbreviations: *FOI* fiber-optic intubation, *LMA* laryngeal mask airway

^a^Data missing = no data found in the database or the corresponding patient records. Where data are missing, percentages are calculated using the total number of available records

Data on intubation difficulty status (easy or difficult) were available for 456 of the 472 patients. Among them, 12.7% (*n* = 58) were described as having had DTI with the corresponding intubation technique used for each case. Intubation with the primary technique failed in 8 patients, whose airways were then managed using either the AirTraq technique (*n* = 4) or asleep intubation with flexible endoscopy (*n* = 4). The airways of 189 (40.0%) patients had been intubated with advanced techniques; the corresponding notes described 6 reasons for use of advanced techniques instead of direct laryngoscopy, including poor mandibular mobility (adequate mouth opening during assessment, but difficulty achieving full mouth opening after anesthetic induction; *n* = 37; 19.6%), cancer growth in the hypopharynx (*n* = 35; 18.5%), trismus (restricted mouth opening at assessment prior to anesthetic induction, *n* = 31; 16.4%), distorted airway anatomy from previous surgery (*n* = 14; 7.4%), short thyromental distance (*n* = 10; 5.3%), and a large tongue (*n* = 10; 5.3%). These 6 reasons accounted for 72.5% of the airways managed by advanced techniques; the reasons for use of advanced airway management techniques in the remaining 52 patients were not specified. Six patients who had initially been scheduled for cancer resection under general anesthesia were found to require tracheostomy before anesthetic induction owing to rapid cancer progression; therefore, airway intubation was not attempted in these patients. No cases of airway loss (cannot intubate and cannot ventilate) were found in the study population.

### Radiation status and airway management characteristics

The mean (± standard deviation) age and BMI in the HNRT group were 58.3 (± 9.2) y and 25.5 (± 4.4) kg/m^2^, respectively. In the control group, the mean age and BMI were 59.1 (± 9.0) y and 25.5 (± 4.5) kg/m^2^. The differences in age (*p* = 0.37) and BMI (*p* = 0.83) between the 2 patient groups were not statistically significant. Table 2 shows associations between radiation status and other covariates. The percentage of patients who had restricted neck range of motion in the HNRT group was significantly higher than in the control group (22.3% vs. 11.0%; *p* = 0.001). In addition, significantly more patients in the HNRT group had advanced-stage cancer than in the control group (51.2% vs. 40.8%; *p* = 0.029). However, the proportion of patients with trismus (*p* = 0.11) and with DAI (*p* = 0.73) did not differ significantly between the 2 groups. Finally, no significant differences were found between the 2 groups in the grade of laryngeal view during tracheal intubation.

**Table 2:** Comparison of patient characteristics by head and neck radiation status

Characteristic	Control N (%)	HNRT N (%)	*P* value
Sex			1.0
Female	30 (12.7)	30 (12.7)	
Male	206 (87.3)	206 (87.3)	
Body mass index (kg/m^2^)			1.0
≤ 18.5	8 (3.4)	9 (3.8)	
18.6–25.0	113 (47.9)	115 (48.7)	
25.1—30.0	79 (33.5)	78 (33.1)	
30.1—35.0	26 (11.0)	25 (10.6)	
35.1—40.0	8 (3.4)	8 (3.4)	
≥ 40.1	2 (0.8)	1 (0.4)	
Mallampati score			0.048
1	42 (18.3)	21 (9.3)	
2	96 (41.7)	109 (48.2)	
3	63 (27.4)	67 (29.6)	
4	29 (12.6)	29 (12.8)	
Mallampati score (1/2 vs. 3/4)			0.59
1/2	138 (60.0)	130 (57.5)	
3/4	92 (40.0)	96 (42.5)	
Mouth opening			0.11
Full	191 (81.3)	176 (75.2)	
Limited	44 (18.7)	58 (24.8)	
Edentulous			0.59
Yes	53 (22.5)	48 (20.4)	
No	183 (77.5)	187 (79.6)	
Neck movement			0.001
Full range	210 (89.0)	181 (77.7)	
Restricted	26 (11.0)	52 (22.3)	
Cancer stage			< 0.0001
T1	28 (13.1)	56 (24.6)	
T2	76 (35.7)	79 (34.6)	
T3	34 (16.0)	58 (25.4)	
T4	75 (35.2)	35 (15.4)	
Intubation status			0.73
Difficult	31 (13.2)	27 (12.2)	
Easy	203 (86.8)	195 (87.8)	
Laryngeal view			0.26
1	80 (58.8)	70 (49.6)	
2	41 (30.1)	58 (41.1)	
3	10 (7.4)	10 (7.1)	
4	5 (3.7)	3 (2.1)	
Laryngeal view (1/2 vs. 3/4)			0.62
1/2	121 (89.0)	128 (90.8)	
3/4	15 (11.0)	13 (9.2)	

Abbreviation: *HNRT* head and neck radiation therapy

Column percentages are provided

The mean (± standard deviation) time interval between completion of HNRT and surgery was 330 ± 475 days and median interval was 134 days. The time interval from HNRT to surgery was not associated with the difficult tracheal intubation (*p* = 0.9363).

### Ease of intubation and patient characteristics

Table 3 displays associations between intubation status and patient characteristics. Data on the intubation status were found for 456 of 472 patients; of these 456 patients, 58 (12.7%) had DTI. The mean (± standard deviation) age of patients who had DTI was 58.2 (± 8.8) y, and the mean age of patients who did not have DTI was 58.8 (± 9.2) y. The mean BMI of patients with DTI was 24.7 (± 4.3) kg/m^2^, and the mean BMI of patients without DTI was 25.6 (± 4.5) kg/m^2^. The 2 groups did not differ significantly in age (*p* = 0.61) or in BMI (*p* = 0.13). Patients who had MP scores of 3 or 4 had significantly higher rates of DTI than did patients with MP scores of 1 or 2 (17.8% vs. 8.7%; *p* = 0.004).

**Table 3:** Comparison of patient characteristics by intubation status

Characteristic	DTI N (%)	Non-DTI N (%)	*P* value
Sex			0.42
Female	9 (16.1)	47 (83.9)	
Male	49 (12.3)	351 (87.8)	
Body mass index (kg/m^2^)			0.23
≤ 18.5	3 (17.6)	14 (82.4)	
18.6–25.0	32 (14.7)	185 (85.3)	
25.1–30.0	19 (12.3)	136 (87.7)	
30.1–35.0	2 (4.1)	47 (95.9)	
35.1–40.0	1 (6.7)	14 (93.3)	
≥ 40.1	1 (33.3)	2 (66.7)	
HNRT			0.73
No	31 (13.2)	203 (86.8)	
Yes	27 (12.2)	195 (87.8)	
Mallampati score			0.032
1	4 (6.5)	58 (93.5)	
2	19 (9.5)	182 (90.5)	
3	22 (17.1)	107 (82.9)	
4	11 (19.6)	45 (80.4)	
Mallampati score (1/2 vs. 3/4)			0.004
1/2	23 (8.7)	240 (91.3)	
3/4	33 (17.8)	152 (82.2)	
Mouth opening			0.53
Full	44 (12.3)	314 (87.7)	
Limited	14 (14.7)	81 (85.3)	
Edentulous			0.42
Yes	10 (10.3)	87 (89.7)	
No	48 (13.4)	310 (86.6)	
Neck movement			0.066
Full range	44 (11.5)	337 (88.5)	
Restricted	14 (19.4)	58 (80.6)	
Cancer stage			0.58
T1	8 (9.6)	75 (90.4)	
T2	23 (15.1)	129 (84.9)	
T3	10 (11.4)	78 (88.6)	
T4	11 (10.8)	91 (89.2)	

Abbreviations: *DTI* difficult tracheal intubation, *HNRT* head and neck radiation therapy

Row percentages are provided

A multivariate logistic regression model was fitted to assess associations between radiation and intubation status after adjustment for neck range of motion and MP score. This analysis revealed no statistically significant association between receiving HNRT and having DTI (Table 4).

**Table 4:** Multivariate logistic regression model showing associations between HNRT and intubation status (DAI vs. non-DAI) adjusted for neck range of motion and Mallampati score

Effect	*P* value	Odds ratio estimate	95% CI^a^	
Treatment (HNRT vs. Control)	0.74	0.907	0.510	1.612
Neck motion (restricted vs. full range)	0.21	1.563	0.773	3.160
Mallampati score (1/2 vs. 3/4)	0.016	0.483	0.268	0.873

Abbreviations: *HNRT* head and neck radiation therapy, *CI* confidence interval

^a^Wald confidence interval

## Discussion

The finding in this study did not reveal the correlation between previous HNRT and DTI in patients with OCC or OPC in spite of significant association between HNRT and restriction of neck range of motion. This can be explained by broadly using flexible endoscopy (35.5%) for tracheal intubation in the studied population, which effectively overcame the influences of restriction of neck range of motion to the performance of tracheal intubation. Nevertheless, our finding does not necessarily mean that the influence of HNRT on tracheal intubation should be underestimated. In this study, 22.3% of patients treated with HNRT had restricted neck movement, whereas only 11.0% of patients in the non-HNRT group did. In addition, although the difference was not statistically significant, more patients in the HNRT group (24.8%) than in the non-HNRT group (18.7%) had trismus. Both restricted neck range of motion and trismus are significant risk factors for difficult direct laryngoscopy. Furthermore, in our clinical experience, trismus and restricted neck range of motion often coexist in patients who develop tissue fibrosis after radiotherapy, posing a significant challenge for both direct laryngoscopy and video laryngoscopy. Because the components of radiation fibrosis syndrome result from the combination of tonic contraction and fibrosis of the muscles of mastication [6], trismus and limited neck range of motion caused by radiation cannot be improved with anesthetic induction or use of a muscle relaxant. This is unlike the trismus induced by cancer pain or inflammation commonly seen in patients who have not undergone radiotherapy, which can often be improved with anesthestics and muscle relaxants. Furthermore, restricted neck range of motion is unlikely to exist in patients without tissue fibrosis unless it is caused by neck-related comorbidities, such as having undergone a neck fusion procedure. No good estimates of the morbidity rate attributable to restriction of neck range of motion after radiotherapy are available, but radiation-induced trismus occurs in up to 45% of patients who receive curative doses of radiation to the head and neck [7]. Thus, having a back-up plan is important when dealing with airways in patients who have undergone HNRT, so that appropriate tools will be available to manage DTI if it arises.

Another significant finding in this study was that MP scores of 3 or 4 were associated with DTI regardless of the technique used. This result is different from a previous report indicating that MP score alone is not significantly correlated with DTI in patients with otherwise normal airway [8]. The discrepancy can likely be attributed to the study populations. The MP scores in our patients reflected the additive effects of oncopathology**,** radiation therapy and baseline condition. A previous study reported that up to 75% of patients developed edema in the head and neck after radiotherapy, and > 50% of patients had edema in the airway [9]. However, the existing grading systems for assessment of airway edema are unreliable and lack consistency [10, 11]. Thus, anesthesiologists who manage such patients’ airways are unlikely to find clinical reports useful in planning airway management. Patterson et al. [11] developed an airway edema rating scale system by measuring intrarater consistency and interrater agreement on the level of airway edema at several anatomic sites. They found that the aryepiglottic folds and arytenoids were the regions most amenable to visual check (Fiber-laryngoscopy) and that intrarater consistency and interrater agreement were low for the base of the tongue. Because radiotherapy-induced airway edema is mostly diffused throughout the area of treatment, the level of edema found in laryngeal or supraglottic areas may reflect edema in the base of the tongue, pharyngeal well, and floor of the mouth. We speculate that moderate edema in any hypopharyngeal or laryngeal site combined with an MP score of 3 or 4 is highly suggestive of DTI. Therefore, a bedside flexible endoscopy airway assessment before anesthetic induction on a patient suspected of airway edema should be incorporated into the routine practice for the clinicians who manage high-risk airways.

It is worth mentioning that the intubation technique used in any given case is based not only on clinical indications but also on the personal preference and skill level of the provider. Therefore, the techniques used in the study might not always directly reflect clinical indications. Furthermore, the outcomes of intubation of difficult airways in patients with OCC or OPC are highly experience-dependent. The techniques and equipment used for intubation are also influenced by local culture or practice; similar cases may be managed differently in different institutions [12, 13]. In this study, the intubations were all performed or supervised by a group of head and neck anesthesiologists who routinely use the techniques described; thus, the results of this study might not be widely applicable to institutions with different practices.

In addition, although the history of HNRT is important to airway management, the anatomic site of the tumor may also affect the airway management. However, the majority of patients (75%) in this study had tumors with local or regional invasion beyond the primary anatomic sites so that grouping the patients by discrete anatomic margins was not feasible. In addition, these cancers induce tissue reaction or inflammation that often extends the mass effect to a much larger area than the tumor itself. Therefore, the causal relationship between primary cancer site and the airway management was not studied.

## Conclusions

In conclusion, history of HNRT does not increase the overall incidences of DTI in patients with OCC or OPC. Mallampati airway assessment may be used as a sensitive tool to predict difficult tracheal intubation in these patients.

## Data Availability

All data generated or analysed during this study are included in this published article and their corresponding figures and tables. Additional data may be available from the corresponding author on reasonable request.

## Abbreviations

ASA score: American Society of Anesthesiologists physical status score
BMI: Body mass index
DTI: Difficult tracheal intubation
HNRT: Head and neck radiation therapy
MP score: Mallampati score
OCC: Oral cavity cancer
OPC: Oropharyngeal cancer
